# Correction: Chen et al. The Protective Effect of Mangiferin on Formaldehyde-Induced HT22 Cell Damage and Cognitive Impairment. *Pharmaceutics* 2023, *15,* 1568

**DOI:** 10.3390/pharmaceutics16121522

**Published:** 2024-11-26

**Authors:** Fan Chen, Na Wang, Xinyan Tian, Juan Su, Yan Qin, Rongqiao He, Xiaping He

**Affiliations:** 1 School of Basic Medical Sciences, Dali University, Dali 671003, China; anlyerxian@foxmail.com (F.C.); wang08140827@163.com (N.W.);; 2 State Key Laboratory of Brain and Cognitive Science, Institute of Biophysics, Chinese Academy of Sciences, Beijing 100045, China; 3 Key Laboratory of Mental Health, Institute of Psychology, Chinese Academy of Sciences, Beijing 100045, China

## Error in Figure

In the original publication [1], there was a mistake in Figure 2 as published. The subfigure of “MGF(200 μM)” in Figure 2 was used incorrectly. The corrected Figure 2 appears below. The authors state that the scientific conclusions are unaffected. This correction was approved by the Academic Editor. The original publication has also been updated.

## Figures and Tables

**Figure 2:**
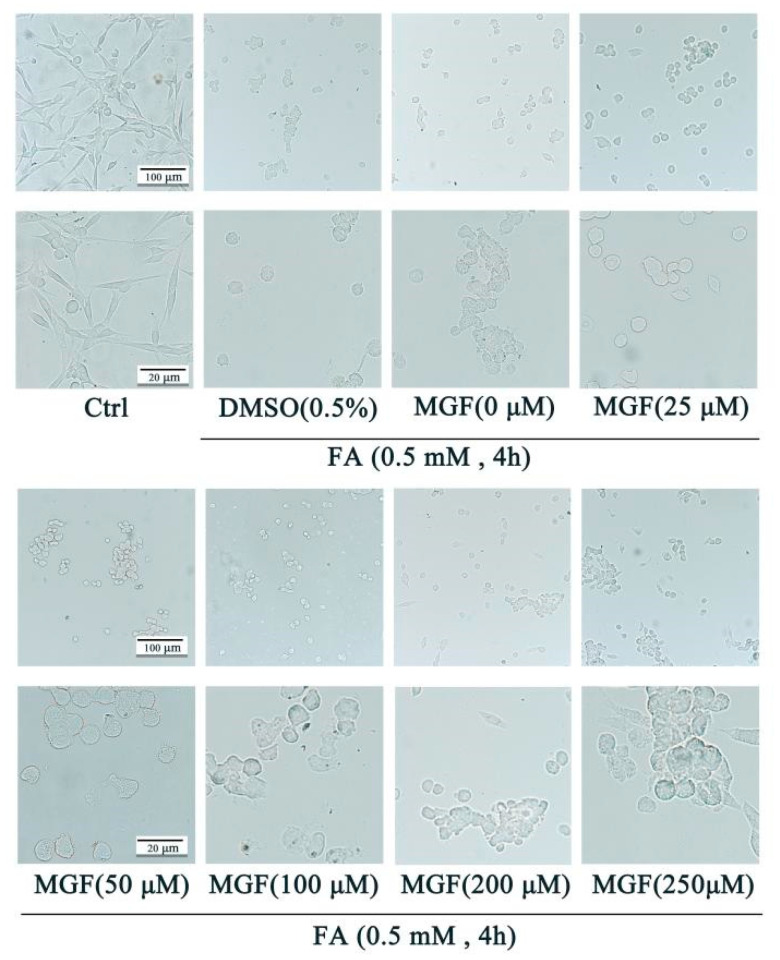
Protective effect of MGF against FA-induced cell morphological changes. HT22 cells were co-treated with MGF (25, 50, 100, 200, 200, and 250 µM) and FA (0.5 mM) for 4 h. Cell morphology was observed using a microscope at different magnifications (**upper** bar: 50 µM; **lower** bar: 25 µM).
